# Variables affecting patellar height in patients undergoing primary total knee replacement

**DOI:** 10.1007/s00264-020-04890-6

**Published:** 2020-12-04

**Authors:** Khaled Hamed Salem, Miten Rajendra Sheth

**Affiliations:** 1 Department of Orthopaedic Surgery, Faculty of Medicine, Cairo University, Cairo, Egypt; 2 Department of Orthopaedic Surgery, RWTH University Aachen, Aachen, Germany; 3 The Knee Clinic, Mumbai, Maharashtra India

**Keywords:** Patellar height, Insall-Salvati ratio, Total knee replacement, Patella Baja

## Abstract

**Background:**

Alteration of patellar height is commonly encountered in total knee arthroplasty (TKA), and failure to address patella baja can result in suboptimal functional outcomes. It may therefore be prudent to evaluate pre-operative patellar height (PPH) and to seek risk factors for patella baja.

**Methods:**

Two hundred eighty-five patients who underwent TKA were included. Patient’s age, gender, body mass index (BMI), and history of prior arthroscopy were recorded. PPH was measured using plateau-patella angle (PPA) as well as the Blackburn-Peel (BP), Caton-Deschamps (CD), and Insall-Salvati (IS) ratios.

**Results:**

The average patients’ age was 71 years with a mean BMI of 30.45. There were 191 female and 94 male patients. One-fourth of the cases had at least one prior knee arthroscopy. Multivariate linear regression analysis identified gender and BMI as variables significantly affecting the IS ratio (*p*: < 0.05). Gender also had a significant correlation with PPA. Male patients were likely to have lower PPA (*p*: < 0.03). Though increasing age had a positive correlation with patellar height, this was not statistically significant. History of prior arthroscopy had no significant effect on any of the four PPH measurements.

**Conclusion:**

Lower patellar height is significantly correlated to male gender and high BMI. We suggest that obese male patients be screened for pre-operative patella baja. This can help in surgical planning and optimizing results in TKA.

## Introduction

Patellar position has a great influence on knee joint biomechanics, and abnormalities in patellar height may affect patellofemoral function [1]. In recent years, patellar height has become an important consideration in evaluating knee conditions and planning treatment, particularly in joint arthroplasty [2, 3]. Several methods have been described to evaluate patellar position, in normal and symptomatic knees, on lateral radiographs. Though no method is perfect, three popular ratios are widespreadly reported in the literature: Insall and Salvati (IS), Blackburne and Peel (BP), and Caton-Deschamps (CD) ratios [4–6]. Recently described, the plateau-patella angle (PPA) has simplified patellar height measurement and is as valid and reproducible as the previously described measurements [7, 8].

Decreased patellar height, known as patella baja or infera, is when the patella is placed too distally relative to the femoral trochlea. Patella baja may occur either due to true shortening of the patellar tendon or elevation of the femoro-tibial joint line (pseudo-patella baja) [9]. This reduction of patellar height is commonly encountered in total knee arthroplasty (TKA), and failure to address it can result in suboptimal functional outcomes [10]. Several studies have focused on patella baja complicating TKA, and the incidence has been reported to be between 25 and 65% [11].

Low pre-operative patellar height (PPH) is an important risk factor for patella baja after TKA [10]. It may therefore be prudent to assess PPH and the variables that may be associated with low PPH.

To the best of our knowledge, this has neither been investigated nor reported to date. The purpose of this study was to report baseline values for pre-operative patellar height and assess the influence of age, gender, body mass index (BMI), and prior arthroscopy on patellar height in patients undergoing primary total knee arthroplasty.

## Materials and methods

### Patient selection

A retrospective consecutive case series was evaluated on digital radiographs from patients who underwent primary unilateral TKA, between September 2014 and July 2015 in a specialized centre for arthroplasty in a university teaching hospital. The indication for surgery in all patients was painful knee osteoarthritis not responding to conservative treatment and significant enough to warrant surgery. Knees with prior history of significant trauma or surgery before TKA, other than arthroscopy, were excluded. Technically poor radiographs with obscured landmarks or alarming malrotation were also excluded. Institutional review board (IRB) approval was obtained. Given the retrospective radiographic evaluation design of this study, an informed patient’s consent was not deemed necessary.

### Image acquisition and assessment

Standard non-weight bearing lateral knee radiographs with a minimum of 30° flexion (up to a maximum of 60°) taken pre-operatively were assessed to measure patellar height. Measurements were made, in millimeters and degrees, using the built-in ruler of the picture archiving and communication system (PACS: MediCAD 3.5, HECTEC GmbH, Germany). Measurements were recorded up to two decimal points. A pilot study, making use of forty pre-operative lateral knee radiographs, was carried out prior to final measurements. The same cohort was then assessed again, at an interval of three weeks from the date of first assessment. The sequence of radiographs was different for the second assessment. These measurements were then reviewed together by the two observers in order to reach a consensus on the technique of measurement.

**Fig. 1:**
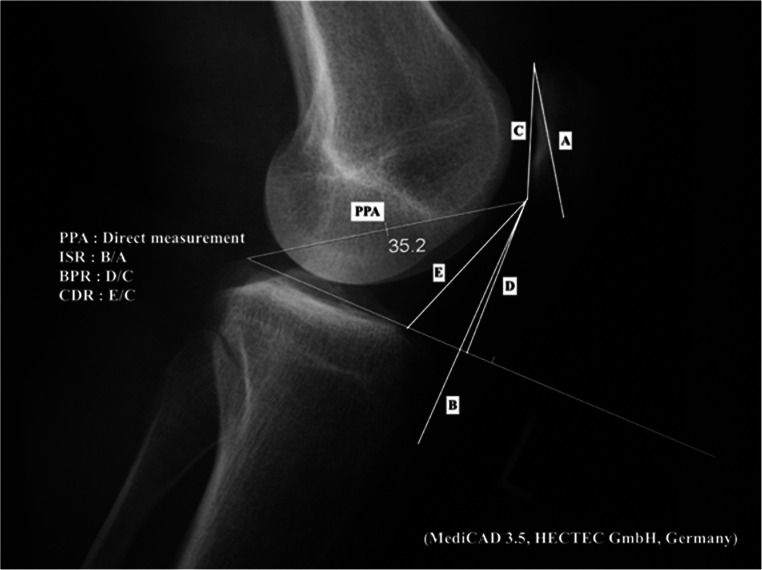
The four parameters used for assessing patellar height

Figure 1 shows the four parameters used for assessing PPH. All indices were measured as described by the primary authors in their original publications [4–7]. Diagonal length of patella (A) and length of the deep surface of patellar tendon (B) were measured to obtain IS ratio (B/A). Length of patellar articular surface (C) and perpendicular distance from the inferior edge of patellar articular surface to the line from tibial plateau surface (D) were used for BP ratio (D/C). Length of patellar articular surface (C) and the distance from the inferior edge of patellar articular surface to the antero-superior angle of tibial plateau surface (E) were used for the CD ratio (E/C). PPA was measured between the line tangential to medial tibial plateau and the line from the posterior extent of medial tibial plateau to inferior articular margin of patella.

Of the total of 314 radiographs reviewed for the current study, 29 (9.2%) were excluded: sixteen radiographs were too rotated; five radiographs had severe arthritic changes with bone loss and/or osteophytes large enough to obscure measurement landmarks; five cases were excluded because the knee had undergone previous surgery other than plain arthroscopy (meniscal surgery, cartilage treatment, partial synovectomy, etc.); two had past history of a fracture of the knee; and one radiograph was excluded because it was done in flexion less than 30 degrees. Thus, 285 radiographs were available for final evaluation and a total of 1710 measurements were made.

### Statistical analysis

Patient’s age, gender, BMI, and history of prior arthroscopy were recorded along with patellar measurements on Excel (Microsoft, Redmond, WA, USA). Descriptive statistics were calculated according to standard methods with range and median or mean and standard deviation. Statistical analysis was performed with SPSS software (SPSS, Chicago, IL). Measurements of the pilot study showed good to excellent inter- and intra-observer reliability and reproducibility (Table 1).

**Table 1:** Pilot measurements

	First measurement				Second measurement			
	ISR	BPR	CDR	PPA	ISR	BPR	CDR	PPA
Inter-observer difference	0.23	0.11	0.17	0.34	0.2	0.07	0.08	0.18
Interclass correlation coefficient	0.83	0.80	0.80	0.67	0.84	0.93	0.87	0.82
95% confidence interval	0.71–0.90	0.65–0.88	0.65–0.88	0.52–0.74	0.56–0.86	0.87–0.96	0.78–0.93	0.69–0.95

*ISR* Insall-Salvati ratio, *BPR* Blackburne-Peel ratio, *CDR* Caton-Deschamps ratio, *PPA* plateau-patella angle

## Results

Two hundred eight-five radiographs met the inclusion criteria. The mean patients’ age was 71 years (median of 73 years, range, 49–90 years). There were 191 female (67%) and 94 male (33%) patients with a female to male ratio of 2:1. One hundred fifty-nine (56%) radiographs were of the right knee. The average patient’s weight was 85.3 kg (range, 53–142 kg), and the mean height was 1.68 m (range, 1.4–1.97 m); hence, the average calculated BMI was 30.45 kg/m^2^ (range: 19.84–49.78 kg/m^2^). Sixty-five patients (23%) had a history of one previous arthroscopy, and one patient had two prior arthroscopies before undergoing TKA.

The average PPH measurements were 1.00 (SD ± 0.16) for IS ratio, 0.84 (SD ± 0.13) for BP ratio, 0.87 (SD ± 0.14) for CD ratio, and 25.41 (SD ± 3.65) for PPA. Depending on the method used, patients were classified as patella baja if their values were two standard deviations below the mean. Table 2 mentions these cut-off points comparing them with the original cut-off values [4–7].

**Table 2:** Cut-off values for patella alta and patella baja

Patellar height measurements	Patella baja		Normal range		Patella alta	
	PPH	Standard	PPH	Standard	PPH	Standard
Insall-Salvati ratio (B/A)	< 0.84	< 0.8	0.84–1.16	0.8–1.2	> 1.16	> 1.2
Blackburne-Peel ratio (D/C)	< 0.7	< 0.8	0.7–0.97	0.8–1.0	> 0.97	> 1.0
Caton-Deschamps ratio (E/C)	< 0.74	< 0.6	0.74–1.01	0.6–1.3	> 1.01	> 1.3
Plateau-Patella angle	< 21.75	< 21	21.75–29.05	21–29	> 29.05	> 29

*PPH* pre-operative patellar height in patients undergoing primary TKA

**Fig. 2:**
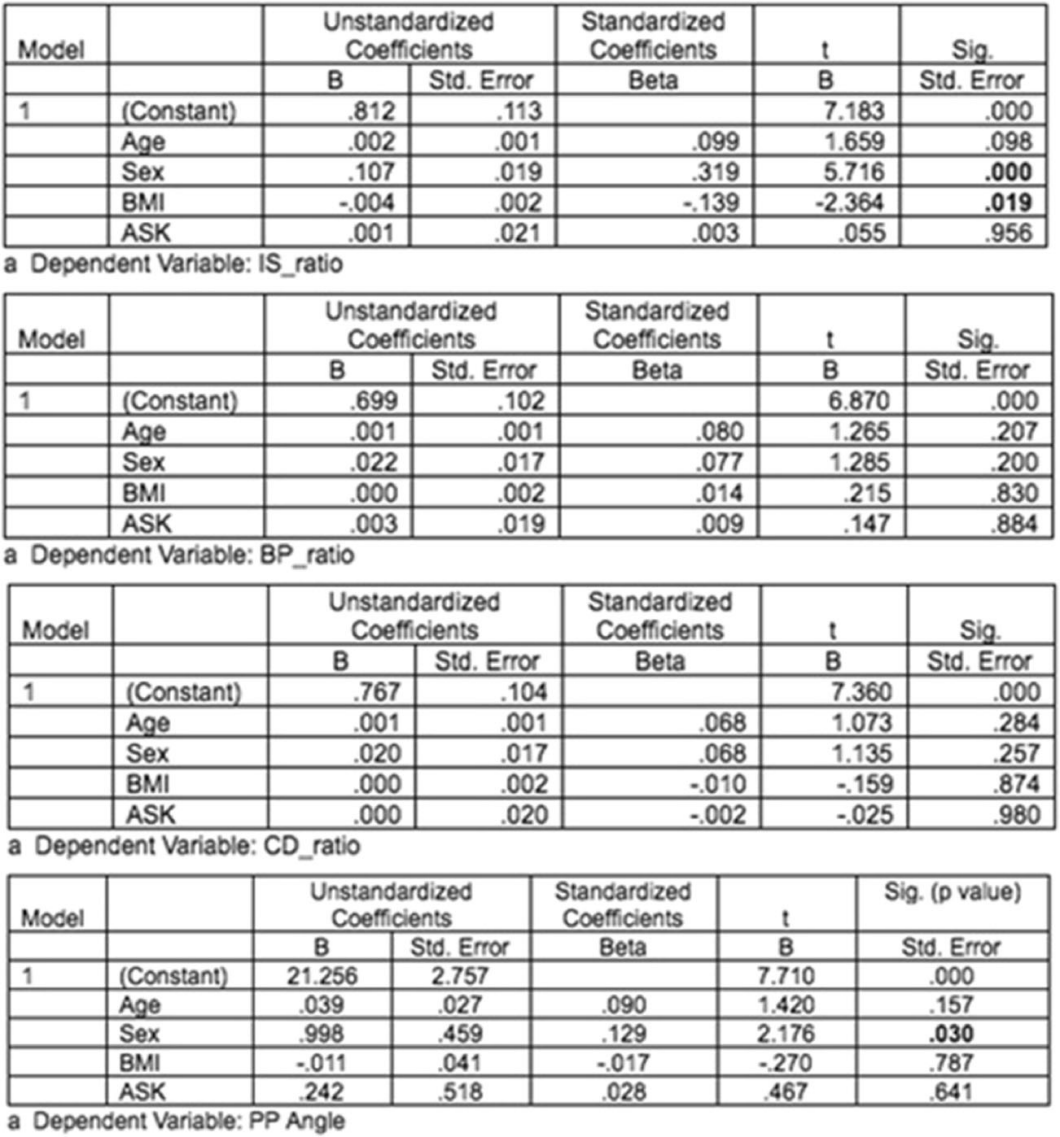
Relation of independent (age, gender, BMI, and history of arthroscopy) and dependent (IS ratio, BP ratio, CD ratio, and PPA) variables

Multivariate linear regression analysis identified gender and BMI as variables significantly affecting patellar height. Gender showed a high correlation with IS ratio. Males are likely to have lower IS ratios compared to females (*p* < 0.001). BMI was associated with a significant negative correlation with IS ratio. Higher BMI is associated with lower IS ratio (*p* = 0.019). Gender also had a significant correlation with PPA. Males were likely to have lower PPA compared to females (*p* = 0.03). Though increasing age has a positive correlation with patellar height measurements, this was not statistically significant. History of prior arthroscopy has no significant effect on any of the four PPH measurements. Figure 2 elucidates the relation of independent (age, gender, BMI and history of arthroscopy) and dependent (IS ratio, BP ratio, CD ratio and PPA) variables along with statistical significance.

## Discussion

Joint line position is linked to functional outcome after primary TKA [12]. Joint line elevation (patella baja) can lead to decreased extensor mechanism power, anterior knee pain due to patellar impingement on the tibial polyethylene insert, and limitation of knee flexion as a result of tightening of the collateral ligaments and diminished femoral rollback [11]. Prevention of post-operative patella baja is the easiest way to avoid these potential problems, and hence, recognition in the pre-operative planning stage is critical [9]. Four measurement methods, including IS ratio, BP ratio, CD ratio, and PPA, have been the most reported diagnostic tools for judging patellar height before and after TKA. All four indices have their own advantages and disadvantages in terms of reliability, reproducibility, and validity, and hence, the surgeon performing TKA may need to use more than one index when evaluating PPH [13–15].

Several studies have reported change in joint line position and its ensuing effects after primary or revision TKA, but none have established baseline values for PPH [12–17]. Our study confirms that PPH value cut-offs (patella baja, normal range, patella alta) for IS ratio, BP ratio, CD ratio, and PPA in patients undergoing primary TKA are not much different from the original reported, established baseline values for the entire population (Table 2). Hence, the traditional cut-off points may also be applicable to this specific subset of patients.

The different association patterns of the different dependent and independent variables are plausible as all four measures of patellar height have their individual limitations. As has been reported in literature, all four indices have their intrinsic differences, and a patient that considered baja on one index may be considered normal on the other index. Hence, the regression analysis was done individually for all the four indices. The current study showed that males tend to have significantly lower pre-operative patellar height (IS ratio and PPA) compared to females. Obese patients with higher BMI also displayed a significantly higher risk of having a lower pre-operative patellar height. Though increasing age has a positive correlation with patellar height measurements, this is not statistically significant. History of prior arthroscopy has no influence on any of the PPH measurements. These results suggest that males and obese patients may be more likely to have a patella baja before TKA.

In addition to low patellar height at pre-operative evaluation, varus/valgus malalignment and excessive collateral ligament laxity are risk factors for patella baja after TKA (higher insert required) [10]. Since PPH is not measured as a routine pre-operatively, there may be some patients with suboptimal functional outcome after TKA (due to patella baja) that may have had a low PPH. Having established that males with higher BMI tend to have a lower pre-operative patellar height, it may therefore be prudent to screen them for patella baja before surgery (especially if accompanied with varus/valgus malalignment or excessive collateral ligament laxity). This can help the surgeon in two ways. Patients with a pre-operative patella baja can be counseled about their risk for post-operative patella baja and ensuing suboptimal functional outcome. Surgeons can plan their operative technique accordingly to minimize the risk of patellar tendon contracture and joint line elevation post-surgery.

There are clearly some limitations to this study. The sample size of our study cohort though large, post-exclusion may not be sufficient to report standard baseline values for PPH in patients undergoing primary TKA. Furthermore, all values were noted by a single observer. Assessor bias is possible, since all measurements after the pilot study were performed by one author alone, relying on previous studies which showed good inter-observer and intra-observer reliability of the four measurement methods used [18–20]. Additionally, an internal pilot study improved the reliability and validity of our measurements. It must be mentioned though that all four indices have inherent methodological measurement difficulties especially when used in patients with osteoarthritic knees. Quadriceps bulk, strength, and degree of relaxation at the time of radiography were not evaluated. But, all radiographs were taken non-weight bearing, so the effect of quadriceps is negated. Patient profile includes ethnicity in addition to age, gender, and BMI. Our cohort included a predominantly Caucasian population. Multivariate linear regression analysis evaluates every independent variable separately, and hence, the influence of age, gender, BMI, and prior knee arthroscopy on PPH can be applied to the entire population, though the effect of ethnicity remains to be tested. We consider the use of normal pre-operative radiographs for evaluation as our strength. These radiographs were of varying quality (e.g., not always true lateral). The use of these radiographs gives a good representation of the imaging encountered in daily practice; therefore, the reliability obtained using these radiographs is more valid than the reliability obtained from perfectly lateral (study-specific) radiographs.

In conclusion, baseline cut-off values for IS ratio, BP ratio, CD ratio, and PPA are the same in patients undergoing primary TKA as for the rest of the population. Pre-operative patellar height is influenced by gender and BMI. Lower IS ratios are significantly correlated to male gender and higher BMI, and lower PPAs are significantly correlated to the male gender. Age and history of knee arthroscopy have no significant influence on pre-operative patellar height. Keeping the findings of this study in mind, we suggest that males with a high BMI be screened for pre-operative patella baja. This can help in surgical planning of primary TKA to avoid post-operative patella baja with suboptimal functional results.
